# Is There Already a Need of Reckoning on Cancer Immunotherapy?

**DOI:** 10.3389/fphar.2021.638279

**Published:** 2021-03-26

**Authors:** Pierpaolo Correale, Francesca Pentimalli, Giovanni Baglio, Marjia Krstic-Demonacos, Rita Emilena Saladino, Antonio Giordano, Luciano Mutti

**Affiliations:** ^1^ Unit of Medical Oncology, Oncology Department, Grand Metropolitan Hospital ‘Bianchi Melacrino Morelli’, Reggio Calabria, Italy; ^2^ Cell Biology and Biotherapy Unit, Istituto Nazionale Tumori, IRCCS, Fondazione G. Pascale, Napoli, Italy; ^3^ Sbarro Institute for Cancer Research and Molecular Medicine, Philadelphia, PA, United States; ^4^ Biomedical Research Center, School of Science, Engineering and Environment, University of Salford, Salford, United Kingdom; ^5^ Tissue Typing Unit, Grand Metropolitan Hospital ‘Bianchi Melacrino Morelli’, Reggio Calabria, Italy; ^6^ Department of Medical Biotechnologies, University of Siena, Siena, Italy; ^7^ Sbarro Institute for Cancer Research and Molecular Medicine, Center for Biotechnology, College of Science and Technology, Temple University, Philadelphia, PA, United States

**Keywords:** immunotherapy, cancer, study design, real world evidences, mesothelioma

The last decades of translational research on cancer immunobiology are allowing the design of profoundly innovative treatments for cancer. Nonetheless, within the scientific community, there is raising concern on the real impact of some of these new possible therapeutic opportunities and on how they are communicated to both health-carers and general public.

Results from randomized clinical trials, which are the gold standard for determining the efficacy of a new treatment, are oftentimes reported as highly successful, despite some significant limitations. A recent survey analyzed over 3,000 randomized controlled trials published in the Journal of the American Medical Association, the Lancet, and the New England Journal of Medicine, to identify low-value medical practices that increase costs without improving care. The authors showed that 396 out of all randomized trials regarding a wide range of medical specialties, including oncology, had not provided results to justify the change of clinical practice as previously supposed (Herrera-Perez et al., 2019).

Also, it is increasingly recognized that some of these trial results should be significantly downsized prior to achieving more convincing validation in the real-world setting (Martini et al., 2020).

Lancet Oncology (The Lancet Oncology, 2018) itself published an authoritative editorial raising a serious warning on the threat posed by the “hype” and information spread across on cancer immunotherapy, and more in general on many new cancer therapies.

Other authors have addressed the risk of miscommunication when poorly reported responses of single patients end up to upstage and replace real achievements based on solid background and validated figures (Nishikawa et al., 2018). This miscommunication leads to high expectations and strong requests from patients and their families, and often clinicians are urged to treat patients with therapies that need a more solid validation.

The hype on some trial results can also affect the drug regulatory practice (Martini et al., 2020); indeed, various cancer drugs have been approved without control arms only in 2019 (CG, 2020). Also concerning is the fact that some studies did not compare new treatments with the best treatment available (Zalcman et al., 2016; Hilal et al., 2019; IASLC, 2020). Other findings show that 67% of the trials which led to anticancer drug marketing authorization by the Food and Drug Administration (FDA) between 2014 and 2019 failed the criteria needed: randomized design, demonstration of survival advantage, appropriate use of crossover, and optimal control arms (Hilal et al., 2020). Similarly, an analysis of pivotal randomized controlled trials of new antitumoral agents approved by the European Medicines Agency (EMA) between 2014 and 2016 concluded that almost half showed a high risk of bias based on their design, conduct, or analysis (Naci et al., 2019).

Other groups have stigmatized that general clinical trials run by not-for-profit organizations are significantly less likely to recommend a new treatment compared to trials conducted by for-profit companies; indeed, 85% of the clinical trials funded by Pharmas report positive results compared to only 50% of government-funded trials (Ridker and Torres, 2006). Analogously, it has been demonstrated that industry-funded trials are up to four times more likely to draw conclusions on support of their products than are independent trials (Sismondo, 2008; Bourgeois et al., 2010; Lundh et al., 2017). Sadly, it has also been red-flagged how the interactions between Pharmas and board members involved into the definition of clinical practice guidelines can also “address” the career of those involved (Desai et al., 2019; Boxe, 2020; Fought et al., 2020; Mitchell et al., 2020).

As predicted (Santye, 2017), one more issue is the recent lowering of the bar for drugs approval by the FDA that certainly played a role to create the current situation (Sherman, 2017).

Authoritative articles have warned about the real size of what at the moment is achievable with this approach (Dechartres et al., 2017; Yarchoan et al., 2017). Unfortunately, as remarked in Lancet Oncology (The Lancet Oncology, 2018), we are all dealing with one of the biggest recent bias in medicine that affects the potential full identification of the actual cost/benefit of cancer immunotherapy itself.

A critical analysis of the overall results of immunotherapies should induce some reflections (Gong et al., 2018): the broad use of surrogate end points and the lack of (plausible) control arms and their high fragility index restrict the real efficacy on overall survival to a few solid tumors, that is, melanoma, non–small cell lung cancer, and renal cell carcinoma, while concern does exist for other common diseases including triple negative breast cancer, small cell lung cancer, head and neck carcinoma, malignant mesothelioma, and other urological malignancies for which further translational-based trials and combination therapies are eagerly required (Gong et al., 2018).

Moreover, the results of immune check point inhibitors (ICIs) on frail patients (even with cancer considered immunotherapy-responsive) are clearly against their use. A recent article has shown that 49% patients hospitalized with ECOG performance status ≥2 (which would have prevented to be enrolled in most clinical trials of ICIs) died during their stay in hospital or within 30 days from discharge, and just 15% of patients were alive 6 months after discharge (Durbin et al., 2021).

Other authors state “Few modern, U.S. FDA-approved immuno-oncology agents have durable survival and response rates that are deemed significant by the American Society of Clinical Oncology value framework” (Dechartres et al., 2017; Ben-Aharon et al., 2018). Notwithstanding, we all are pressured to use immunotherapy approaches. We have read “wonder drug” up in a petition (Robson, 2016) to ask health systems to provide these extremely expensive drugs, whereas other groups publish a book to declare that “immunotherapy is the end of cancer” (Maio and Minoli, 2018).

Eventually, other authors conclude that the conflict of interest represented by the links between doctors and pharmaceutical companies (Dechartres et al., 2017; Santye, 2017; Sherman, 2017; Yarchoan et al., 2017; Mitchell et al., 2018) could also contribute to explain the miscommunications on novel treatments like immunotherapy (Kaestner et al., 2017).

The need of a more transparent collaboration between clinical scientists and pharmaceutical companies (in particular those involved in cancer immunotherapy) has been recently raised to avoid biases in the conduction of the randomized clinical trials even leading to registration by regulatory bodies (Schirrmacher et al., 2020). With this regard, Hasting Center Report by De Jesus-Morales and Vinay Prasad includes an article entitled “*Closed Financial Loops: When They Happen in Government, They’re Called Corruption; in Medicine, They’re Just a Footnote*” (De Jesus-Morales and Prasad, 2017).

Moreover, other evidences show that trial results are often either not communicated or reported with significant delay. This occurs despite the 2017 rule, enacted by the National Institutes of Health (NIH) and the FDA, which clarified law expectations and penalties for those failing to disclose promptly trial results (Piller, 2020).

Even though we believe that most clinical scientists act according to the highest ethical standards, there is no doubt that more transparency on the nature and entity of any sort of conflict of interest could avoid any doubt on all the process.

Eventually, one should consider what happens with “orphan” tumors where the urgency of better treatments clashes with the lack of proper preclinical studies. The result of this discrepancy leads to the exploitation of the pipelines rather than building a strong preclinical background (Elmhirst and Brown, 2020).

Among others, this is what is happening with mesothelioma (a pleural cancer mainly due to asbestos exposure) where after a long period of time when tremelimumab and pembrolizumab were hailed as a revolution for this tumor on the basis of earlier small studies (“*the syrup* that melts tumors” (Fondazione Buzzi Unicem, 2016), until the results of randomized clinical trials and real-world evidence with overall survival as end points have demonstrated their inefficacy (Maio et al., 2017; Popat et al., 2020) and pointed out how the outcome was rather biased by performance status real-world evidence (Ahmadzada et al., 2020; Lau et al., 2020).

More recently, a combination of ICIs has been demonstrated to top standard chemotherapy for mesothelioma. However, the comparison control arm was not the best treatment known (Zalcman et al., 2016; IASLC, 2020). If one compares the survival of ICIs with the best treatment, this latter is still superior.

Likewise, other empirical approaches without neither background nor solid study design and control arm have been proposed to be the new frontline therapy for this orphan disease (Barbarino et al., 2020; de Gooijer and Burgers, 2020).

**FIGURE 1:**
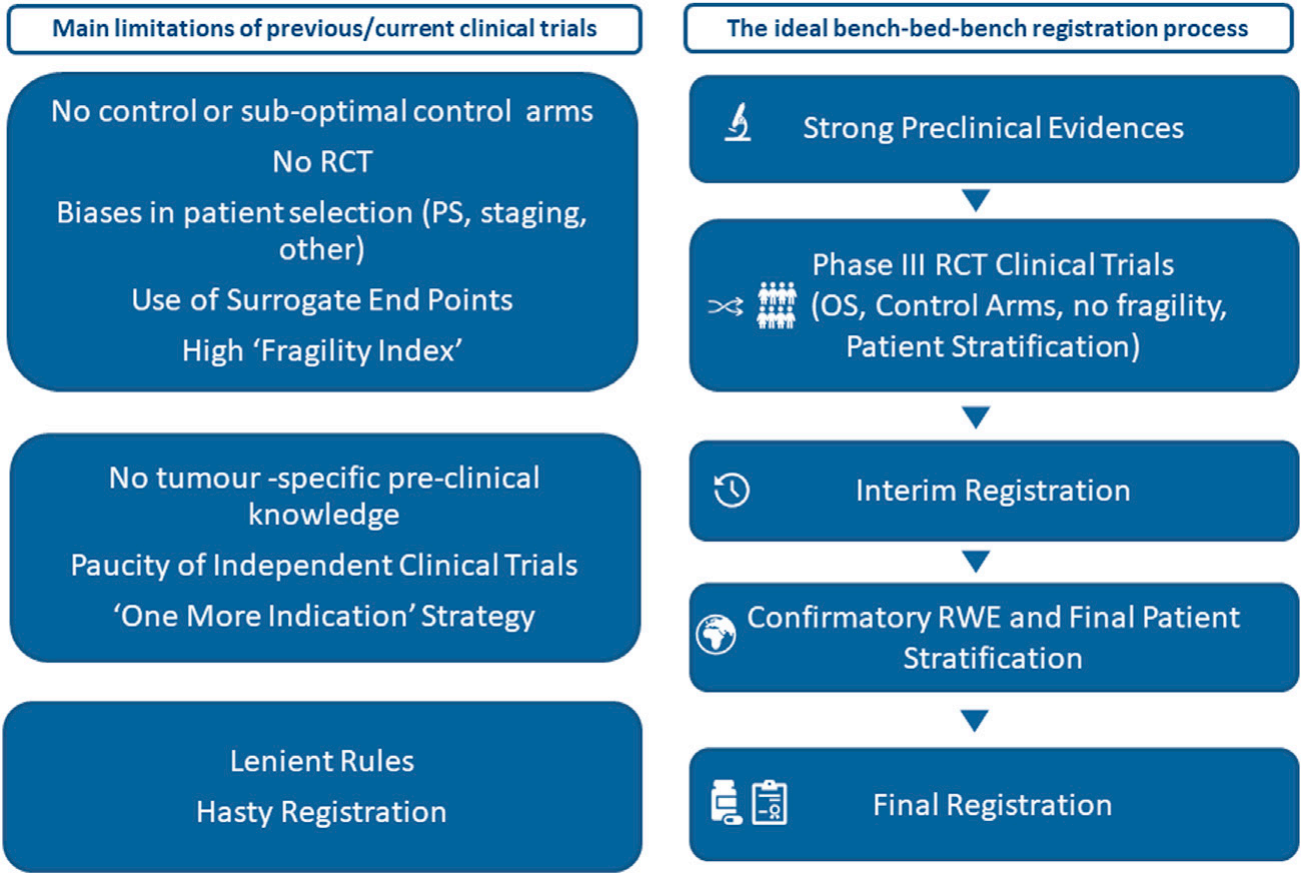
Main limitations of many current clinical trials and an ideal bench–bed–bench registration process. OS: overall survival; PS: performance status; RCT: randomized clinical trials; RWE: real-world evidence.

Therefore, the lack of a strong tumor-specific rationale, the characteristic of the patient enrolled (most of them in very good performance status) and the suboptimal end points and control arms together with high fragility index of many of these trial (Bomze et al., 2020; Bomze and Meirson, 2019) influence the success of the trials and may lead to the registration of therapies despite uncertain efficacy to justify their toxicity and exorbitant economic costs (Schirrmacher et al., 2020). Moreover, several cautionary tales suggest the results for clinical trials should not be set in stone before being validated by everyday performance by real-world evidence (Van Cutsem, 1999; Muhsin et al., 2003; Correale et al., 2016; Proto et al., 2019; Martini et al., 2020). We hope that a more science-based approval process will inspire the approval of new immunotherapies for cancer (Figure 1) and create the conditions for an even synergism between the need of results quality and validation and the registration of new drugs for cancer immuneotherapy.
